# Regional Personalized Electrodes to Select Transcranial Current Stimulation Target

**DOI:** 10.3389/fnhum.2013.00131

**Published:** 2013-04-22

**Authors:** Franca Tecchio, A. Cancelli, C. Cottone, L. Tomasevic, B. Devigus, G. Zito, Matilde Ercolani, F. Carducci

**Affiliations:** ^1^Laboratory of Electrophysiology for Translational neuroScience (LET’S) – ISTC – CNR, Department of Neuroscience, Fatebenefratelli HospitalRome, Italy; ^2^Department of Neuroimaging, IRCCS San Raffaele PisanaRome, Italy; ^3^Institute of Neurology, Cattolica del Sacro Cuore UniversityRome, Italy; ^4^Department of Neuroscience and Imaging, G. d’Annunzio University of Chieti – PescaraItaly; ^5^Computing and Mathematics, Science and Technology, University of PlymouthPlymouth, Devon, UK; ^6^Department of Psychology, Sapienza University of RomeRome, Italy; ^7^Associazione Fatebenefratelli per la Ricerca, Department of Clinical Neuroscience, Fatebenefratelli HospitalRome, Italy; ^8^Neuroimaging Laboratory, Department of Physiology and Pharmacology, Sapienza University of RomeRome, Italy

**Keywords:** transcranial current stimulation, neuronavigation, motor cortex, somatosensory cortex, personalized stimulation target, customized electrodes

## Abstract

**Rationale:** Personalizing transcranial stimulations promises to enhance beneficial effects for individual patients.

**Objective:** To stimulate specific cortical regions by developing a procedure to bend and position custom shaped electrodes; to probe the effects on cortical excitability produced when the properly customized electrode is targeting different cortical areas.

**Method:** An *ad hoc* neuronavigation procedure was developed to accurately shape and place the personalized electrodes on the basis of individual brain magnetic resonance images (MRI) on bilateral primary motor (M1) and somatosensory (S1) cortices. The transcranial alternating current stimulation (tACS) protocol published by Feurra et al. (2011b) was used to test the effects on cortical excitability of the personalized electrode when targeting S1 or M1.

**Results:** Neuronal excitability as evaluated by tACS was different when targeting M1 or S1, with the General Estimating Equation model indicating a clear tCS Effect (*p* < 0.001), and *post hoc* comparisons showing solely M1 20 Hz tACS to reduce M1 excitability with respect to baseline and other tACS conditions.

**Conclusions:** The present work indicates that specific cortical regions can be targeted by tCS properly shaping and positioning the stimulating electrode.

**Significance:** Through multimodal brain investigations continuous efforts in understanding the neuronal changes related to specific neurological or psychiatric diseases become more relevant as our ability to build the compensating interventions improves. An important step forward on this path is the ability to target the specific cortical area of interest, as shown in the present pilot work.

## Introduction

Electrophysiology can be used to bridge the gap between the changes in brain activity after damage and the construction of efficacious compensating interventions. Recent advancements of transcranial current stimulation (tCS) protocols involved the induction of proper excitation/inhibition effects to selected regional targets. For example, the orbitofrontal regions delta-like anodal stimulation to potentiate slow-wave sleep activity, enhancing declarative memory (Marshall et al., 2004), lesional anodal, and contralesional cathodal stimulation of hemispheric homologous areas in unilateral strokes to counterbalance excessive inhibition exerted by the contralesional areas, thus supporting functional recovery (Stagg et al., 2012). Finally, left anodal and right cathodal dorsolateral prefrontal cortices in depression to re-establish proper hemispheric excitability features (Shafi et al., 2012).

Along this line, the ability to focus the stimulation on specific target areas is crucial; with the present work we developed a procedure to properly shape the stimulating electrode. Specifically, we aimed at targeting the bilateral primary sensory vs. motor areas planning in the near future to implement a compensating intervention to relief fatigue in multiple sclerosis patients. Our working hypothesis is that selectively enhancing S1 excitability, we can further improve the functional connectivity between parieto-frontal networks, already known to be reinforced by tCS over SM1, without enhancing M1 excitability (Polania et al., 2011). In fact, in MS fatigued patients M1 excitability is higher than in non-fatigued and in healthy controls (Nielsen and Norgaard, 2002; Thickbroom et al., 2006) and we have indications of impaired communication between S1 and M1 (Dell’Acqua et al., 2010; Tomasevic et al., 2013).

The modulation of cortical excitability generated by tCS can be focused by means of proper sizing and positioning of the stimulating electrode. In the motor system, it was compared the tCS effects on the central representations of two muscles, the first dorsal interosseus (FDI) and abductor digiti minimi (ADM) (Nitsche et al., 2007). By measuring the peak-to-peak amplitude of motor evoked potentials (MEPs), differential effects were documented by a focal increase of MEPs from each muscle depending on tCS electrode positioning. The protocol proposed in the present work requires less focused stimulation than Nitsche’s, where a discrimination of M1 neuronal pools controlling the two hand muscles was sought. In fact, we intended to stimulate the entire motor and somatosensory regions. However, while the positioning of tCS electrodes on M1 can be guided by TMS coil location, a neuronavigation system is mandatory when selectively stimulating S1 or M1, since they are contiguous in the anterior-posterior direction. Thus, we developed a proper procedure exploiting modern frameless stereotaxic systems to navigate across the subject’s brain structures, via three-dimensional magnetic resonance imaging (MRI) data. The required high spatial precision, with accuracy in the millimeters range as documented for TMS (Sparing et al., 2008), can be transferred to tCS electrode positioning as well. In our experimental setup, a topographically precise determination of the central sulcus is mandatory in order to shape and place the S1 and M1 electrodes, when targeting areas that are contiguous in the anterior-posterior direction.

The need to develop a tCS electrode that selectively targets bilateral S1 or M1, led us: (1) to test the feasibility of shaping and positioning personalized electrodes for targeting a distinct cortical area; (2) to prove that this personalized electrode allows to differentially modulate neuronal excitability when targeting S1 in respect with M1. For this second aim, we exploited a recently introduced procedure (Feurra et al., 2011b). The excellent opportunity documented by Feurra et al. (2011b) is to probe tACS induced online effects by concurrent transcranial magnetic stimulation (TMS). This is a promising tool to empower other neuromodulation paradigms, by defining relevant features through a quick and efficient protocol. At the same time, tACS stimulation – at proper frequencies of the applied current – is able to modify the excitability of visual (Kanai et al., 2008, 2010) and somatosensory systems (Feurra et al., 2011a,b) as well as to improve the performance in cognitive tasks (by stimulating current modulated in a wide frequency range, called Random Noise Stimulation – tRNS, Fertonani et al., 2011).

## Materials and Methods

### Participants

Five healthy, right-handed volunteers (four females, one male; age range 25–56 years) with normal neurological examination and medical history were included in the study, after signing the informed consent approved by the Ethics Committee of “San Giovanni Calibita” Fatebenefratelli Hospital. None of them had been taking psychoactive drugs for the past 6 months.

### Regional personalized electrodes’ shaping

A few days before the experimental session, each subject underwent a structural brain MRI exam with a 1.5 T scanner (Achieva, Philips Medical Systems, Best, Netherlands), provided with a 33 mT/m gradient amplitude, an online 2D/3D geometric distortion correction and an 8-channel head phased-array coil. T1-3D Fast Field Echo sequences with full brain coverage (MPRAGE, TR/TE/FA = 8.6 ms/4 ms/8°; 170 contiguous sagittal slices 1.2 mm thick without gap, mtx1922) were acquired.

**Figure 1:**
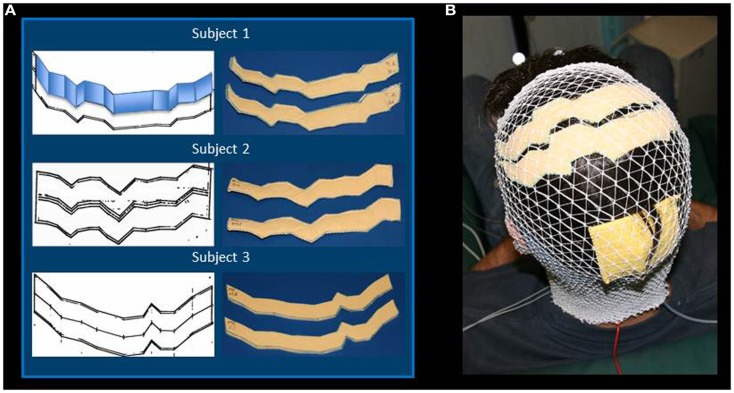
**Regional Personalized Electrode shaping**. The sponge electrodes are shaped, for each subject, in two steps: first, drawing the left and right central sulci on a piece of paper using SoftTaxic software from a volumetric MRI; second, fitting the central sulcus by 2 cm-width parallelograms (an example is reported for Subject 1). For each S1 and M1 electrode, the shape is than drawn on and cut out of two sponge sheets; the two sheets are sewed together to allow the insertion of conductive material **(A)**. The stimulating electrodes are positioned by proper neuronavigation procedure (reference landmark is visible frontally), while the reference electrode is positioned according to the alignment used by Feurra et al. (2011a). Electrodes are secured through an elastic cotton cap **(B)**.

The MRI data were elaborated by the SofTaxic Neuronavigation System ver. 2.0 (www.softaxic.com, E.M.S., Bologna, Italy) in order to guide the stereotaxic procedure for the electrodes’ personalization. Specifically, while the subject sat on a comfortable chair, the central sulcus was identified on his/her 3D-rendered brain, and its projection upon the scalp was traced on a paper sheet – well stabilized on the scalp – by means of a pen fixed to the SofTaxic stylus, properly recalibrated. From each individual trace of the central sulcus, the personalized S1 and M1 sponge electrodes were modeled: the central sulcus was fitted into segments so that the electrode was shaped into parallelograms of 2 cm widths, starting from Cz, and maintaining the same length in the left and right hemispheres. Left and right lateral borders were defined in order to set the effective area to 35 cm^2^ (Figure 1A). Finally, each electrode was completed by sewing together two identical sponge surfaces at their edges and then inserting a copper wire between the sponges for current transmission (Figures 1A,B).

### Personalized electrode positioning

SofTaxic navigation allowed placing the sponge electrodes in line with the central sulcus, anteriorly for M1 and posteriorly for S1. The two electrodes were previously soaked in a saline solution and affixed to the subject’s head with a conductive gel. The reference electrode was a 7 × 5 cm rectangle positioned above Oz.

### Transcranial alternating current stimulation

Sinusoidal stimulations at 10 or 20 Hz were delivered through a current stimulator charged with a battery (Eldith Stimulator by NeuroConn, Ilmenau, Germany) at a peak-to-peak intensity of 1000 μA. The efficacious current superficial density was thus 10.1 μA/cm^2^. Impedances were below 10 kΩ throughout the stimulations. In addition to S1 or M1 tACS, Sham stimulation was provided (4 s of active stimulation at the beginning and the end of each 1.5 min session). Each stimulation block lasted 1.5 min, was randomly delivered across subjects and intermingled by more than 3 min between each session. At debriefing, no subject reported to feel any difference across tCSs.

### Transcranial magnetic stimulation

Transcranial magnetic stimulation to probe differential effects of S1 and M1 tACS targeting Single-pulse TMS was performed through a standard focal coil (the diameter of each wing was 70 mm) connected with a SuperRapid module (The Magstim Company Ltd, Whitland, UK). TMS MEPs were recorded on the opponens pollicis (OP) of the left and right hands by surface electrodes in a belly tendon montage (2.5 cm apart).

**Figure 2:**
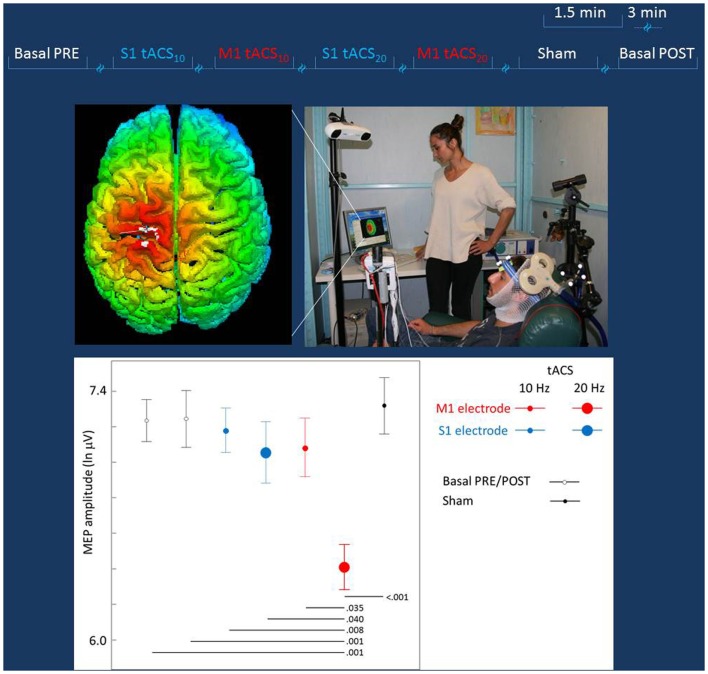
**Experiment to probe differential effects of stimulation target**. Top lines: representation of the stimulation blocks, lasting 1.5 min each and intermingled by more than 3 min. The blocks (Sham, S1, or M1 tACS at 10 or 20 Hz) were randomly delivered across subjects. Middle Left: real time scalp projection of the TMS coil position onto the 3D-rendered cortical surface overlying the tCS electrodes to probe online the different effects on the cortical excitability as induced by the stimulation of the two cortical targets (the cross indicated the center of the coil over the central sulcus). Middle Right: experimental setup of the TMS session. The TMS focal coil overlies the tCS personalized M1 electrode; the TMS coil position is stabilized by a mechanical arm that is digitized once the OP hot-spot is identified and monitored throughout the experiment duration. Bottom: the MEP amplitude in the different tCS conditions. *Post hoc* comparisons are reported for significant differences.

The hot-spot of the right OP muscle was identified (TMS coil positioned above personalized tCS electrodes). Thereafter, the coil position was maintained by a support arm that was digitized and monitored throughout the entire session with the SofTaxic neuronavigator (Figure 2). TMS was applied at the intensity adjusted to produce OP MEP amplitudes of about 1–1.2 mV in basal conditions (i.e., TMS applied through personalized electrodes, without tCS). While each tCS (tACS or Sham) was active, TMS stimuli were elicited with an inter-stimulus interval randomly changing between 5 and 7 s (about 15 repetitions for each tCS).

### Statistical analysis

If necessary, after checking the distribution shape of the MEP amplitude, a suitable transformation was applied in order to achieve a better approximation to gaussianity and a good control of outliers. The estimating procedure often adopted in neurostimulation studies (Feurra et al., 2011a) uses Analysis of Variance (ANOVA) for repeated measures on the mean of the single trial peak-to-peak MEP amplitudes obtained during stimulations and during baseline. Together with that, in the present work we have also considered the within-subject variability, extending a procedure introduced in a previous study of our laboratory (Tecchio et al., 2008), where we documented that no inter-trial correlations occur among various MEPs, thus making this model appropriate to evaluate both the final values and the intra-subject variability. We took into account all MEP repetitions, so to properly weight means corresponding to lower or higher inter-trial variability, applying a General Estimating Equation model with single trial MEP amplitude as the dependent variable and tCS (S1 tACS10Hz, S1 tACS20Hz, M1 tACS10Hz, M1 tACS20Hz, Sham) as predictors. The alpha-inflation due to multiple comparisons was faced according to Sidak’s procedure.

## Results

The mean across subjects TMS intensity was 74 ± 5.4% of maximal stimulator output. The MEP amplitude distribution definitely differed from a Gaussian one and a good fit was obtained by natural logarithmic transformation. In baseline conditions, the mean MEP latency was 23.6 ± 0.8 ms and the mean MEP intensity was 1436 μV (obtained by exponential back-transformation of the mean of logarithm-transformed MEP amplitudes). No association between the order of MEP collection and its amplitude was observed (Pearson’s correlation *p* = 0.607). The oneway ANOVA design indicated a strong tCS effect [*F*(6, 489) = 4.442, *p* < 0.001], with *post hoc* comparison showing selectively M1 20 Hz tACS differing from all other conditions (*p* < 0.01 for all conditions but S1 20Hz tACS *p* = 0.040 and M1 10 Hz tACS *p* = 0.035). No other condition differed from any other. The General Estimating Equation model indicated a clear tCS Effect [tCS factor Wald Chi-square = 2554.417, df = 4, *p* < 0.001; Figure 2], with *post hoc* comparisons showing solely M1 tACS20Hz reduced with respect to baselines (Pre and Post) and from all other transcranial stimulation conditions (Figure 2).

## Discussion

This work tested the feasibility of a procedure to personalize specific cortical targeting by tCSs, providing the procedural details to shape and position the stimulating electrode based on the three-dimensional reconstruction of structural MRI of each subject. The MRI-guided neuronavigation system was also used to precisely locate the electrodes onto the subject’s scalp. The documented electrode-dependent cortical excitability modulation refines previous evidence that it is possible to focus the effects of tCS by properly shaping and positioning the electrodes to target a region of interest of the cerebral cortex.

EEG and MEG data gathered in our laboratory showed signs of a disruption of primary somatosensory network patterning in MS (Tecchio et al., 2008; Dell’Acqua et al., 2010) and MS fatigue (Tomasevic et al., 2013). Linking this functional indications to cortical atrophy of the parietal lobe in patients affected by multiple sclerosis fatigue (Pellicano et al., 2010), we are aiming at modifying a transcranial direct current stimulation (tDCS) intervention on SM1 which was able to enhance endurance to fatigue in healthy people (Cogiamanian et al., 2007). So, first of all we decided to selectively target S1 instead of SM1. Neither electrophysiological (Dell’Acqua et al., 2010) nor neuroimaging data (Pellicano et al., 2010) provided evidence for mono-hemispheric prevalence in MS fatigue. Furthermore, the indication of interhemispheric functional connectivity reduction with fatigue (Peltier et al., 2005) suggests intervening with stimulations that support interhemispheric dynamical balance. Hence, we decided to target S1 bilaterally as a whole, covering the body representation of upper and lower limbs (Marshall et al., 2004). Once decided to target bilateral S1 to relieve MS fatigue, the first step was to build a procedure for the electrode proper shaping and positioning, as we describe in the present paper.

By applying the same stimulation parameters, current intensity, alternating frequency, electrodes’ area as in Feurra et al. (2011a), M1 excitability was expected to increase during tACS at 20 Hz. On the contrary, the M1 20 Hz tACS induced a clear decrease of excitability; it has been observed (Moliadze et al., 2012) that inhibitory vs. excitatory effects were induced by tACS delivered at different current densities (for 0.4 vs. 1 mA across the same electrode). Furthermore, Salvador et al. (2010) estimated the magnitude of the current density in a realistic conductive model observing definite non-homogeneity at the interface between gray matter and cerebrospinal fluid, which produced stronger effects (about twice as strong) at the depth of cortical sulci beneath the central region than at the border of the electrode. In conclusion, although other factors – the most important being bilateral stimulation – cannot be excluded at the origin of our results, our main explanation is that a less efficacious current reached M1’s pyramidal neurons in our experiment than in Feurra’s. We are building a devoted experiment to verify such a possibility because to relief fatigue in MS patients an enhancement of the excitability of the target region S1 is required.

Although the data leading to target selection indicated that a bilateral stimulation is required, the comparison with electrodes used by other authors could be very useful in future experiments to better understand specific effects introduced by the customized electrodes developed herein. Furthermore, bilateral stimulation might have induced different effects from Feurra et al. (2011a) (compensatory and/or excitatory/inhibitory effects) and devoted protocol could be very interesting to comprehend how to intervene on homologous areas to sustain their balance, this latter becoming more and more evident as a key aspect of control system functioning. Finally, although our main interest is on S1 effects, the protocol proposed by Feurra et al. (2011a) provided the opportunity to test directly the effects on M1 excitability, while no direct measure was collected in relation to S1 excitability.

The present pilot work indicates – for the first time outside primary motor area where TMS coil position can guide the electrode placing – the feasibility of a procedure aimed at shaping and positioning with millimetric precision customized electrodes for tCS, potentially enhancing the ability to properly build neuromodulation interventions to compensate neurological or psychiatric induced neuronal dysfunctions.

## Conflict of Interest Statement

The authors declare that the research was conducted in the absence of any commercial or financial relationships that could be construed as a potential conflict of interest.
